# High-Quality Genome Sequence of the Highly Resistant Bacterium *Staphylococcus haemolyticus*, Isolated from a Neonatal Bloodstream Infection

**DOI:** 10.1128/genomeA.00683-17

**Published:** 2017-07-20

**Authors:** Farideh Hosseinkhani, Mohammad Emaneini, Willem van Leeuwen

**Affiliations:** a Department of Innovative Molecular Diagnostics, University of Applied Sciences Leiden, Leiden, The Netherlands; b Department of Microbiology, School of Medicine, Tehran University of Medical Sciences, Tehran, Iran

## Abstract

Using Illumina HiSeq and PacBio technologies, we sequenced the genome of the multidrug-resistant bacterium *Staphylococcus haemolyticus*, originating from a bloodstream infection in a neonate. The sequence data can be used as an accurate reference sequence.

## GENOME ANNOUNCEMENT

Among the coagulase-negative staphylococcal (CoNS) species, *Staphylococcus haemolyticus* plays an important role as an opportunistic pathogen in hospital-acquired infections worldwide (1). It is frequently isolated from blood cultures, especially from patients in neonatal intensive care units (2). The incidence of multidrug-resistant *S. haemolyticus* with the new staphylococcal cassette chromosome *mec* (SCC*mec*) is increasing. This bacterium has been considered an important reservoir for resistance genes that can be transmitted to other staphylococcal species, such as the highly virulent *Staphylococcus aureus* (2, 3). The structure of its genome is poorly understood. In order to obtain knowledge about the highly resistant character of this species and the possibility for the transmission of resistance genes, we sequenced the genome of a multidrug-resistant *S. haemolyticus* strain.

The strain (SH06) originated from a bloodstream infection of a 2-year-old patient admitted to the Children’s Medical Center (Tehran, Iran) in May 2013. This strain was identified based on morphology, biochemistry, and matrix-assisted laser desorption ionization–time of flight mass spectrometry (MALDI-TOF MS [Vitek]) analysis and finally confirmed by *nuc* gene PCR (4). DNA was extracted from strain SH06 obtained after overnight incubation on sheep’s blood agar at 37°C, using the UltraClean microbial DNA isolation kit (Mo Bio Laboratories, Inc.) according to the manufacturer’s instructions.

Sequencing was performed using the paired-end method with the Illumina HiSeq2500 system. A FASTQ sequence file was generated using the Illumina Casava pipeline version 1.8.3. The data collected from the PacBio RS instrument were processed and filtered using the SMRT analysis software suite. The continuous long-read (CLR) data were filtered by read length (>35), subread length (>35), and read quality (>0.75). The final PacBio read statistic was provided as Enclosure. The quality of the Illumina FASTQ sequences was enhanced by trimming off low-quality bases using the program bbduk, which is part of the BBMap suite version 34.46. The quality-filtered sequence reads were assembled into a number of contig sequences. The analysis was performed using ABySS version 1.5.1. The contigs were linked and placed into super scaffolds based on the alignment of the PacBio CLR reads. Alignment was performed with BLASR (5). The orientation and order of, as well as the distance between, the contigs were estimated from the alignment. This analysis was performed using the SSPACE-LongRead scaffolder version 1.0 (6). The gapped regions within the super scaffolds were (partially) closed in an automated manner using GapFiller version 1.10 (7). The method takes advantage of the insert size between the Illumina paired-end reads.

Genome annotation was performed on the assembled contig and scaffold sequences using the Prokka prokaryotic genome annotation system (Prokka version 1.6).

The sequence analysis revealed the size of the strain SH06 genome to be 2,501,418 bp, fragmented into 4 scaffolds (*N*_*50*_ of 1,555,818 bp; G+C content of 32.65). The genomic data generated provide an accurate reference sequence of *S. haemolyticus* that can provide a leap forward in comparative genomic analysis.

### Accession number(s).

This whole-genome shotgun project has been deposited at DDBJ/ENA/GenBank under the accession number NFUG00000000. The version described in this paper is the first version, NFUG01000000.
